# Gut microbiome signatures predict cognitive impairment in older cancer survivors

**DOI:** 10.1007/s11357-025-01917-x

**Published:** 2025-11-01

**Authors:** Brandi C. Miller, Jayden A. Haggler, Diptaraj S. Chaudhari, Rohit Shukla, Vivek Kumar, Sidharth P. Mishra, Michal M. Masternak, Peter Holland, Corinne Labyak, Adam Golden, Mariana Dangiolo, Andrea Y. Arikawa, Judyta Kociolek, Amoy Fraser, Cynthia Williams, Marc Agronin, Mariolga Aymat, Warren Pledger, Hariom Yadav, Shalini Jain

**Affiliations:** 1 USF Center for Microbiome Research, Microbiomes Institute, University of South Florida Morsani College of Medicine, Tampa, FL USA; 2 Center for Excellence in Aging and Brain Repair, University of South Florida Morsani College of Medicine, Tampa, FL USA; 3 Department of Neurosurgery and Brain Repair, University of South Florida Morsani College of Medicine, Tampa, FL USA; 4 Burnett School of Biomedical Sciences, College of Medicine, University of Central Florida, Orlando, FL USA; 5 Department of Head and Neck Surgery, Poznan University of Medical Sciences, Poznan, Poland; 6 Department of Neuroscience, Schmidt College of Medicine, Florida Atlantic University, Boca Raton, FL USA; 7 Department of Nutrition and Dietetics, University of North Florida, Jacksonville, FL USA; 8 Department of Internal Medicine, University of Central Florida College of Medicine, Orlando, FL USA; 9 Clinical Research Unit, Division of Research, Florida Atlantic University, Boca Raton, FL USA; 10 School of Global Health Management and Informatics, University of Central Florida, Orlando, FL USA; 11 Behavioral Health, MIND Institute, Miami Jewish Health, Miami, FL USA; 12 Tampa General Hospital Cancer Institute, Tampa, FL USA

**Keywords:** Microbiome, Aging, Gut, Brain, Cancer, Cognition

## Abstract

Cancer treatments are improving, and the population of cancer survivors is steadily increasing. However, many survivors experience long-term side effects, including chemobrain and other age-related geriatric disorders like cognitive impairment (CI), severely impacting their quality of life. Emerging studies suggest that the gut microbiome plays a central role in cognitive health. However, the long-term effects of cancer treatments on the microbiome, and how these changes impact cognitive health in survivors, remain largely unknown. Shotgun metagenomic data from 150 older adults (≥ 60 years old, including 49 cancer survivors and 101 controls) from the Microbiome in Aging Gut and Brain (MiaGB) consortium revealed that *Tyzzerella*, *Eggerthella lenta*, and *Bacteroides vulgatus* were specific markers of the cancer survivor gut and could differentiate cancer survivorship in this cohort. Microbiome signatures were distinct in cancer survivors with CI compared to those without and differed from those seen in non-cancer individuals with CI. Bacterial taxa including *Streptococcus thermophilus* and *Firmicutes bacterium* CAG 114 were significantly reduced in cancer survivors and strongly associated with CI. Importantly, metabolic pathway analysis revealed that microbial neurotransmitter synthesis was significantly depleted in the gut of cancer survivors, suggesting a mechanistic link to CI. Our results suggest that microbiome signatures predict cancer survivorship and the risk of CI in older adults, potentially by depleting neurotransmitter synthesis in the gut. These findings aid in establishing the role of the microbiome in predicting cancer survivorship and CI risk, which is valuable in the development of novel therapies to support the growing population of cancer survivors.

**Graphical Abstract:**

**Figure:**
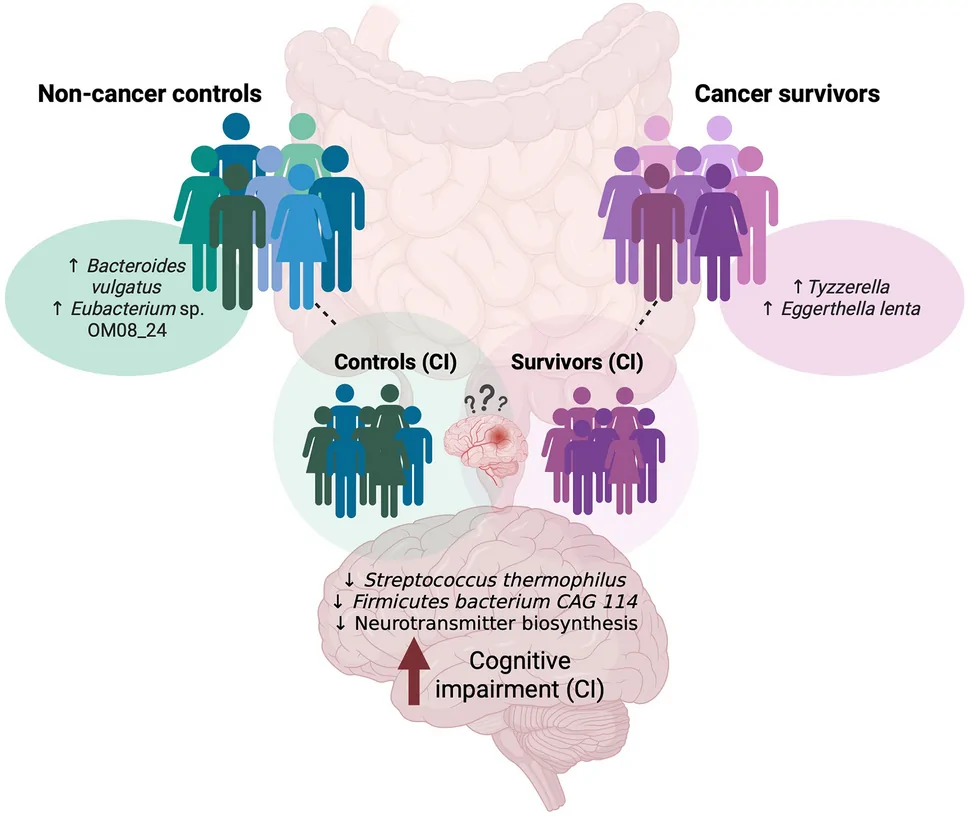

**Supplementary Information:**

The online version contains supplementary material available at https://doi.org/10.1007/s11357-025-01917-x.

## Introduction

Cancer treatments, including chemotherapy, radiotherapy, immunotherapy, or combination regimens, have become increasingly effective, leading to higher survival rates over the past few decades [1]. This represents a major milestone for the medical, scientific, and public health communities. However, while many side effects subside after treatment, cancer therapies often cause severe adverse effects during treatment, and a substantial number of survivors continue to experience these effects long-term. Many of these conditions are associated with impairments in gut and brain health, including diarrhea, indigestion, nausea, vomiting, appetite loss, constipation, and “chemo brain” [2]. Moreover, brain dysfunctions further increase the risk of developing cognitive impairment (CI), dementia, and other neurodegenerative diseases. Collectively, these issues significantly diminish the quality of life (QoL) in cancer survivors. A recent meta-analysis reported that approximately one-third of breast cancer survivors experience long-term CI following treatment (in some cases, more than 10 years) [3], while another study found that CI was accelerated in survivors during a 20-year follow-up, compared to non-cancer controls [4]. Similar deficits in cognitive function have been reported in survivors of other cancer types, including head and neck, colorectal, prostate, and testicular cancers [5–8]. However, the precise reasons for the long-term side effects of cancer treatments and their underlying mechanisms remain elusive, thus hindering the development of effective and implementable remedies to combat these cancer treatment-induced ailments.

Abnormalities in the gut microbiome are a suspect in the development of cancer treatment-related long-term side effects. Multiple emerging studies, including ours, indicate that perturbations in the gut microbiome contribute to gut and brain health as well as cancer pathogenesis and the efficacy of cancer treatments in both mice and humans [9–12]. The composition and function of the gut microbiota significantly differ in many human diseases, including aging, and also significantly impact gut-brain functions [13, 14]. Our lab and others have shown that older adults and those with mild cognitive impairment (MCI) and/or dementia have a unique gut microbiota, compared to their controls [10, 15–17]. Emerging studies have also suggested that cancer survivors have reduced microbiome diversity and harbor significantly different gut microbiome signatures, such as decreased abundances of *Firmicutes*, *Selenomondales, Veilloneliaceae*, *Faecalibacterium*, and *Intestinibacter*, and enrichment of Proteobacteria, compared to their non-cancer controls [18–20]. These abnormal microbiome signatures in cancer survivors of various cancer types, including non-Hodgkin’s lymphoma or other blood cancers, were linked with changes in microbial and energy metabolism, activation of the immune system, and poor gut and psychosocial health [18, 20]. In fact, specific bacterial taxa, such as *Intestinibacter*, *Lachnospiraceae*, *Ruminococcaceae*, and *Bacteroides*, were significantly correlated with diarrhea or poor psychosocial health [2, 20]. Despite these preliminary findings and attempts to link intestinal microbiome structure and function with gut and brain health in cancer survivors, there are still significant gaps in our understanding of these connections. In particular, the long-term compositional and functional changes in microbiome signatures as a result of cancer treatments, specifically in older adult cancer survivors, remain unknown. Furthermore, it remains elusive how structural and functional changes in the gut microbiota of cancer survivors are linked with their cognitive function. While a few studies have assessed microbiome signatures in cancer survivors and have discussed their implications on cognitive health, these studies do not investigate functional changes in the microbiota to explore the potential mechanisms connecting the gut microbiome with cognitive function [20–23]. To the best of our knowledge, there are no studies that have determined the potential of the microbiome and its metabolic pathways as biomarkers to differentiate older adults cancer survivors from non-cancer survivor controls as well as those with CI and/or dementia.

Herein, to address these clinically relevant and scientifically critical knowledge gaps, we conducted a pilot analysis using the data and samples from our ongoing multi-site clinical study—the Microbiome in aging Gut and Brain (MiaGB) consortium. We have performed whole genome metagenomics analyses to show that the gut of cancer survivors harbors a unique microbiome signature, with predicted changes in metabolic functions, compared to non-cancer controls, and is linked with cognitive function and the potential to predict cancer survivorship and CI.

## Methods

### Human cohorts

Data and samples for this study were obtained from the MiaGB consortium cohort. The MiaGB study is ongoing and recruits community-dwelling older adults (≥ 60 years) from five sites in Florida, with an aim of understanding the gut-brain axis in aging [24]. The demographics of all study participants (*n* = 150) are shown in Supplementary Table S1. The study excludes individuals who have a history of brain- or gut-related surgeries within the last 5 years, diagnosis with neurological disorders such as epilepsy, Parkinson’s disease, and amyotrophic lateral sclerosis, use of antibiotics within the last 30 days, prevalence of gastrointestinal side effects, such as diarrhea and vomiting in the last 30 days, and a history of inflammatory bowel disease or other related gastrointestinal conditions. For these analyses, we excluded participants who were missing data for age, cancer history, and cognitive function. All participants provided written informed consent, and all study procedures were conducted in accordance with the protocols approved by the Institutional Review Board of the University of South Florida.

To create our cohorts, we first stratified participants into a non-cancer control group or a cancer survivor group, based on their self-reported cancer history (Table 1). Cancer survivors were study participants who had previously been diagnosed with cancer of any type at least 5 years prior to the study and had completed their treatment. Individuals who had cancer at the time of enrollment were excluded from the study. The non-cancer control group included participants who had never been diagnosed with any type of cancer. Data regarding cancer survivorship were retrieved from demographic surveys completed by participants during their initial study visit. We further stratified participants based on cognitive function using scores from the Montreal Cognitive Assessment (MoCA), as described below (Table 2). Pairwise comparisons were made between non-cancer controls and cancer survivors and were also performed based on cancer survivorship and cognitive function together.

**Table 1:** Demographics of study participants stratified based on cancer survivorship

Demographic	Controls	Cancer survivors
Number of participants in cohort	101	49
Female (%)	69 (68.32%)	31 (63.27%)
Male (%)	32 (31.68%)	18 (36.73%)
Age (years)	71.51 ± 0.73	76.55 ± 1.20***
BMI (kg/m^2^)	26.59 ± 0.54	27.35 ± 0.75
Ethnicity (%)		
Not Hispanic or Latino Hispanic or Latino Unknown race or unreported	81 (80.20%) 13 (12.87%) 7 (6.93%)	41 (83.67%) 4 (8.165%) 4 (8.165%)
Race (%)		
White Black or African American Asian Unknown race or unreported	91 (90.1%) 4 (3.96%) 3 (2.97%) 3 (2.97%)	46 (93.88%) 0 (0.0%) 2 (4.08%) 1 (2.04%)

All data are presented as mean ± SEM or frequency (%), as appropriate

*BMI* Body mass index, *SEM* Standard error of the mean, ***p < 0.001

**Table 2:** Participant demographics, stratified based on previous cancer status and cognitive function

Demographic	Non-cancer, no CI	Non-cancer, CI	Cancer, no CI	Cancer, CI
Number of patients in cohort	64	37	29	20
Female (%)	48 (75%)	21 (56.8%)	20 (68.97%)	11 (55%)
Male (%)	16 (25%)	16 (43.2%)	9 (31.03%)	9 (45%)
Age (years)	70.22 ± 0.91	73.76 ± 1.15*	74.17 ± 1.37	80.0 ± 1.97*^##&^
BMI (kg/m^2^)	26.05 ± 0.58	27.52 ± 1.10	28.96 ± 1.00	25.02 ± 0.96
Ethnicity (%)				
Not Hispanic or Latino Hispanic or Latino Unknown ethnicity/unreported	51 (79.69%) 9 (14.06%) 4 (6.25%)	30 (81.08%) 4 (10.81%) 3 (8.11%)	25 (86.2%) 2 (6.9%) 2 (6.9%)	16 (80%) 2 (10%) 2 (10%)
Race (%)				
White Black or African American Asian Unknown race/unreported	59 (92.2%) 2 (3.1%) 1 (1.6%) 2 (3.1%)	32 (86.5%) 2 (5.4%) 2 (5.4%) 1 (2.7%)	28 (96.6%) 0 (0.0%) 0 (0.0%) 1 (3.4%)	18 (90%) 0 (0.0%) 2 (10%) 0 (0.0%)
MoCA Score	27.72 ± 0.15	21.76 ± 0.57	27.90 ± 0.26	22.35 ± 0.47

All data are presented as mean ± SEM or frequency (%), as appropriate. *p < 0.05, compared to non-cancer, no CI group; ^##^*p* < 0.01, compared to non-cancer, CI group; ^&^*p* < 0.05, compared to cancer, no CI group

*BMI* body mass index, *CI* cognitive impairment, *MoCA* Montreal Cognitive Assessment, *SEM* standard error of the mean

### Stool sample collection

Stool samples were collected using an in-house stool sample collection kit, which we have implemented for this clinical study and other ongoing studies at our lab. During their study visit, participants were handed a stool collection kit and asked to collect the sample within 24 h. The kits include all necessary items as well as directions for proper stool collection. We also explained stool collection procedures to participants during their study visit. Upon return of the kits, stool samples were immediately aliquoted and stored at − 80 °C until analysis.

### Cognitive function assessment

Cognitive function was measured using the MoCA. The MoCA is a well-validated questionnaire for determining cognitive function in older adults (55 years of age and older), with a maximum score of 30. Participants with a MoCA score of 26 or higher were considered cognitively healthy, while those with a score < 26 were considered to have MCI (score 18–25) or dementia (score < 17) [25]. We classified all participants with MCI or dementia as part of our CI group.

### Metagenomic shotgun sequencing

Whole-genome shotgun sequencing was performed using our previously established protocols [26]. Briefly, genomic DNA was extracted from stool samples (~ 150 mg) using the QIAamp PowerFecal Pro DNA kit (Qiagen), according to the manufacturer’s instructions. Extracted DNA was quantified using the Nanodrop spectrophotometer (ThermoFisher Scientific, USA) and Qubit dsDNA HS assay kit (ThermoFisher Scientific, USA). We used 150 ng of DNA for library preparation using Illumina DNA Prep and the (M) Tagmentation kit (Illumina, Inc., 5200 Illumina Way, San Diego, CA, USA), according to the manufacturer’s instructions. Furthermore, we used sample-specific and unique IDT for Illumina-Nextera DNA UD Indexes. Sequencing was performed using the Illumina NextSeq1000 and Illumina NextSeq 1000/2000 P2 reagents (300 cycles) v3 reagent cartridge (Illumina, Inc., San Diego, CA, USA). All data were stored in the BaseSpace cloud and analyzed according to the bioinformatics and statistical analyses described below.

### Bioinformatics analyses

Data from our metagenomic shotgun sequencing were analyzed using the Yet Another Metagenomic Pipeline (YAMP) workflow, which utilizes tools from the bbmap suite to de-duplicate, trim, and decontaminate the metagenomic reads, as well as FastQC to visualize raw and QC-filtered reads [26, 27]. We used MicrobiomeAnalyst (https://www.microbiomeanalyst.ca/) to determine α-diversity measures, including Shannon and Simpson indices, and observed operational taxonomic units (OTUs). Additionally, we used HUMAnN 3.0 (HMP Unified Metabolic Analysis Network), which is comprised of the MetaPhlAn and ChocoPhlAn pangenome databases, to perform pathway analysis and determine the metabolic capacity of the stool microbiota [28]. MetaPhlAn was used to predict and identify all microbial species within each sample and perform nucleotide level pangenome mapping in accordance with the ChocoPhlAn database, which provides filtered metagenomic reads [28]. This genome mapping resulted in organism-specific gene hits, which are utilized by the core HUMAnN algorithm. Remaining unmapped reads from ChocoPhlAn were aligned against the Universal reference database (UniRef50) using the Diamond tool [29]. Alignments from both databases were input into the HUMAnN algorithm to determine the abundances of metabolic pathways and gene families. Identified microbes and predicted pathways from these metagenomics analyses were subject to the statistics described below.

### Statistical analyses

We assessed both α- and β-diversity for the transkingdom (archaea, bacteria, and viruses together), as well as individually at the bacterial and viral levels. α-Diversity measures were visualized using column charts or boxplots, while β-diversity (microbiome diversity across groups) was analyzed using principal component analysis (PCA) based on Euclidean distances. Each dot represents the sample from one participant. Stacked and individual column graphs show relative taxonomic abundances at the phylum, family, genus, and species levels for bacteria, family and species levels for viruses, and at the species level for archaea. The shared and unique archaeal, bacterial, and viral taxa as well as metabolic pathways were calculated and plotted using interactiVenn (http://www.interactivenn.net). Furthermore, we calculated linear discriminant analysis (LDA) scores using the LDA effect size (LefSe) algorithm on the Galaxy platform (https://huttenhower.sph.harvard.edu/galaxy/) and show significant taxa at the genus and species levels [30]. R scripts were used to generate heat maps (package ggplot2), random forest analyses (RFA; packages randomForest, ggplot2, lattice, and caret), and correlation plots (package corrplot) for clustering, prediction, and correlation analyses, respectively, for bacterial taxa and metabolic pathways. Microsoft Excel was used to calculate fold change values for up- and downregulated bacterial taxa as well as predicted metabolic pathways from our HUMAnN 3.0 analysis. We used GraphPad Prism v9.0 to construct bar charts and volcano plots showing these changes in taxonomy or metabolic pathways, respectively. Pearson’s correlation coefficient was calculated to determine significant correlations between bacterial or viral taxa, metabolic pathways, and MoCA score. We plotted these correlations using hierarchical clustering in Python or constructed two-variable correlation plots (including linear regression line and 95% confidence intervals (95%CI)) in GraphPad Prism. Area under the curve (AUC) and 95%CIs for prediction analysis were calculated using multiple logistic regression analysis in GraphPad Prism, and we constructed receiver operating characteristic (ROC) curves. Statistical analyses were performed using GraphPad Prism v9.0 or Microsoft Excel. We used unpaired *t*-tests and Welch’s *t*-test (assumes unequal variance), as appropriate, for pairwise comparisons of α-diversity indices, relative abundances, and expression of metabolic pathways between the groups. *p* < 0.05 was considered statistically significant for all analyses. On volcano plots, values greater than -log_10_(0.05) were considered statistically significant.

## Results

### The gut of older cancer survivors harbors a distinct microbiome compared to non-cancer controls

**Fig. 1:**
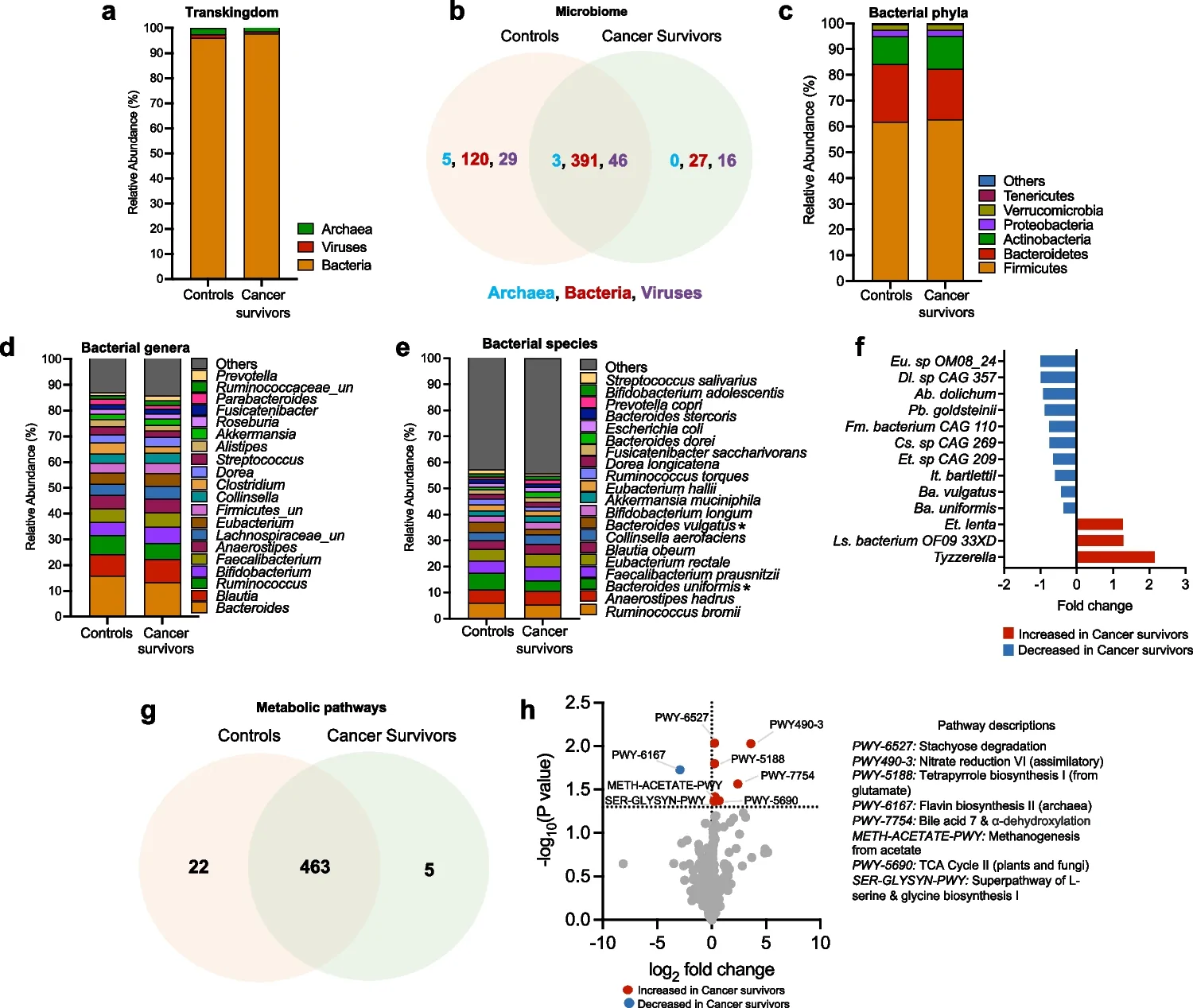
The gut of older adult cancer survivors harbors a distinct microbiome structure and function compared to non-cancer controls. **a** Transkingdom abundances of bacteria, viruses, and archaea in cancer survivors and non-cancer controls. **b** Venn diagram showing shared and unique archaea, bacteria, and viruses in the two groups. **c** Relative abundances of major bacterial phyla. **d** Relative abundances of major bacterial genera. **e** Relative abundances of major bacterial species. *Ba. uniformis* and *Ba. vulgatus* were significantly depleted in the gut of cancer survivors compared to controls. **f** Significantly increased (red) and decreased (blue) bacterial genera and species in the cancer survivor gut, expressed as fold change compared to controls. Fold change values less than -log_10_(0.05) were considered statistically significant. **g** Venn diagram depicting the unique and shared predicted metabolic pathways in the two groups. **h** Volcano plot illustrating the increased (red) and decreased (blue) metabolic pathways in cancer survivors compared to non-cancer controls. Values are expressed as the difference in the log_2_ fold change, and values less than -log_10_(0.05) were considered statistically significant. Abbreviations: *Ab. dolichum*, *Absiella dolichum*; *Ba. uniformis*, *Bacteroides uniformis*; *Ba vulgatus*, *Bacteroides vulgatus*; *Cs.* sp. CAG 269, *Clostridium* sp. CAG 269; *Dl.* sp. CAG 357, *Dialister* sp. CAG 357; *Et. lenta*, *Eggerthella lenta*; *Et.* sp. CAG 209, *Eggerthella* sp. CAG 209; *Eu.* sp. OM08_24, *Eubacterium* sp. OM08_24; *Fm. bacterium* CAG 110, *Firmicutes bacterium* CAG 110; *Ls. bacterium* OF09 33XD, *Lachnospiraceae bacterium* OF09 33XD; *Pb. goldsteinii*, *Parabacteroides goldensteinii*. *p < 0.05, **p < 0.01, ***p < 0.001, ***p < 0.0001, according to unpaired t-test (with Welch’s correction, as appropriate, for unequal variances)

Using metagenomic shotgun sequencing on stool DNA, we observed that the gut of older adults with a history of cancer (survivors, *n* = 49) harbors a distinct microbiome compared to non-cancer controls (*n* = 101) (Fig. 1). Although transkingdom analysis revealed there were no significant differences in the relative abundances of bacteria, viruses, or archaea between these groups (Fig. 1a), we found that there were 27 bacteria and 16 viruses unique to cancer survivors at the species level (Fig. 1b). There were no significant differences in transkingdom β-diversity (Supplementary Figure S1a) or α-diversity measures, including the Shannon index, Simpson index, Chao1, and observed OTUs (Supplementary Figure S1b-e). Additionally, there were no significant differences in β- or α-diversity measures of the bacteriome between cancer survivors and non-cancer controls (Supplementary Figure S1f-i). Interestingly, we show that the relative abundance of Gram-negative taxa was slightly decreased in the cancer survivor gut (31.54%) compared to controls (33.89%), but the difference was not statistically significant (Supplementary Figure S2a). We did not detect any significant differences in phylogenetic abundances of bacteria at the phylum level (Fig. 1c), the Firmicutes:Bacteroidetes (F/B) ratio (Supplementary Figure S2b), or in the top 15 families (Supplementary Figure S2c). However, the abundances of family *Erysipelotrichaceae* and genus *Tyzzerella* were significantly increased in the gut of cancer survivors compared to non-cancer controls (Fig. 1d; Supplementary Figure S2d, e). Furthermore, species-level analysis revealed that *Bacteroides (Ba.) uniformis* and *Ba. vulgatus* were among the top 20 species and were significantly depleted in the cancer survivor gut (Fig. 1e, Supplementary Figure S2h, i). We also calculated the fold change of the significantly different bacterial taxa to determine the magnitude of their differences in cancer survivors compared to controls (Fig. 1f). *Tyzzerella*, *Lachnospiraceae (Ls.) bacterium* OF09 33XD, and *Eggerthella (Et.) lenta* were significantly enriched in cancer survivors (Fig. 1f, Supplementary Figure S2e-g). On the other hand, there were ten bacterial species, including *Ba. uniformis*, *Ba. vulgatus*, *Intestinibacter bartlettii*, and *Et. sp.* CAG 209, with a significantly lower abundance in cancer survivors compared to controls (Fig. 1f, Supplementary Figure S2h-q). These data suggest that there are unique bacterial signatures present in the gut of older cancer survivors compared to their non-cancer controls.

Furthermore, our whole genome metagenomic sequencing allows us to quantify diversity and abundance measures of other microbes, including viruses and archaea. There were no significant differences in viral α-diversity measures, including the Shannon and Simpson indices and observed OTUs (Supplementary Figure S3a-c). Additionally, we did not observe any significant variations in the phylogenetic abundances of the major virus families between the groups (Supplementary Figure S3d). However, at the species level, *Lactococcus phage P335 *sensu lato was significantly depleted in the gut of cancer survivors compared to controls (Supplementary Figure S3e, f). The abundances of the major archaea species were similar in both groups (Supplementary Figure S3g). Overall, these data suggest that the gut microbiota composition of cancer survivors is unique, at the transkingdom, bacterial genera, and bacterial and viral species levels, compared to that of their non-cancer controls.

### The gut microbiome of cancer survivors is functionally distinct

One major mechanism by which the gut microbiota impacts human health is through their metabolic activities and the production of metabolites. Thus, we used our metagenomics data to elucidate the metabolic activities of the microbiome. Although we did not observe significant clustering of metabolic pathways, according to PCA (Supplementary Figure S4), there were 22 metabolic pathways unique to non-cancer controls and 5 pathways unique to cancer survivors (Fig. 1g; Supplementary Table S2). Using volcano plot analyses to depict the log_2_ fold change, we found that metabolic pathways related to stachyose degradation (PWY-6527), assimilatory nitrate reduction VI (PWY490-3), tetrapyrrole biosynthesis II from glutamate (PWY-5188), bile acid 7 and α-dehydroxylation (PWY-7754), methanogenesis from acetate (METH-ACETATE-PWY), tricarboxylic acid (TCA) cycle II of plants and fungi (PWY-5690), and superpathway of L-serine and glycine biosynthesis I (SER-GLYSYN-PWY) were significantly increased in the cancer survivor gut, while flavin biosynthesis II from archaea (PWY-6167) was decreased (Fig. 1h). Supplementary Table S3 shows the full names, descriptions, and log_2_ fold change values for all significantly increased and decreased pathways in the cancer survivor gut compared to controls. Collectively, these data indicate that the gut of older cancer survivors has distinct metabolic functions compared to that of non-cancer controls.

### Distinct microbiota and their metabolic pathways can differentiate cancer survivorship in older adults

**Fig. 2:**
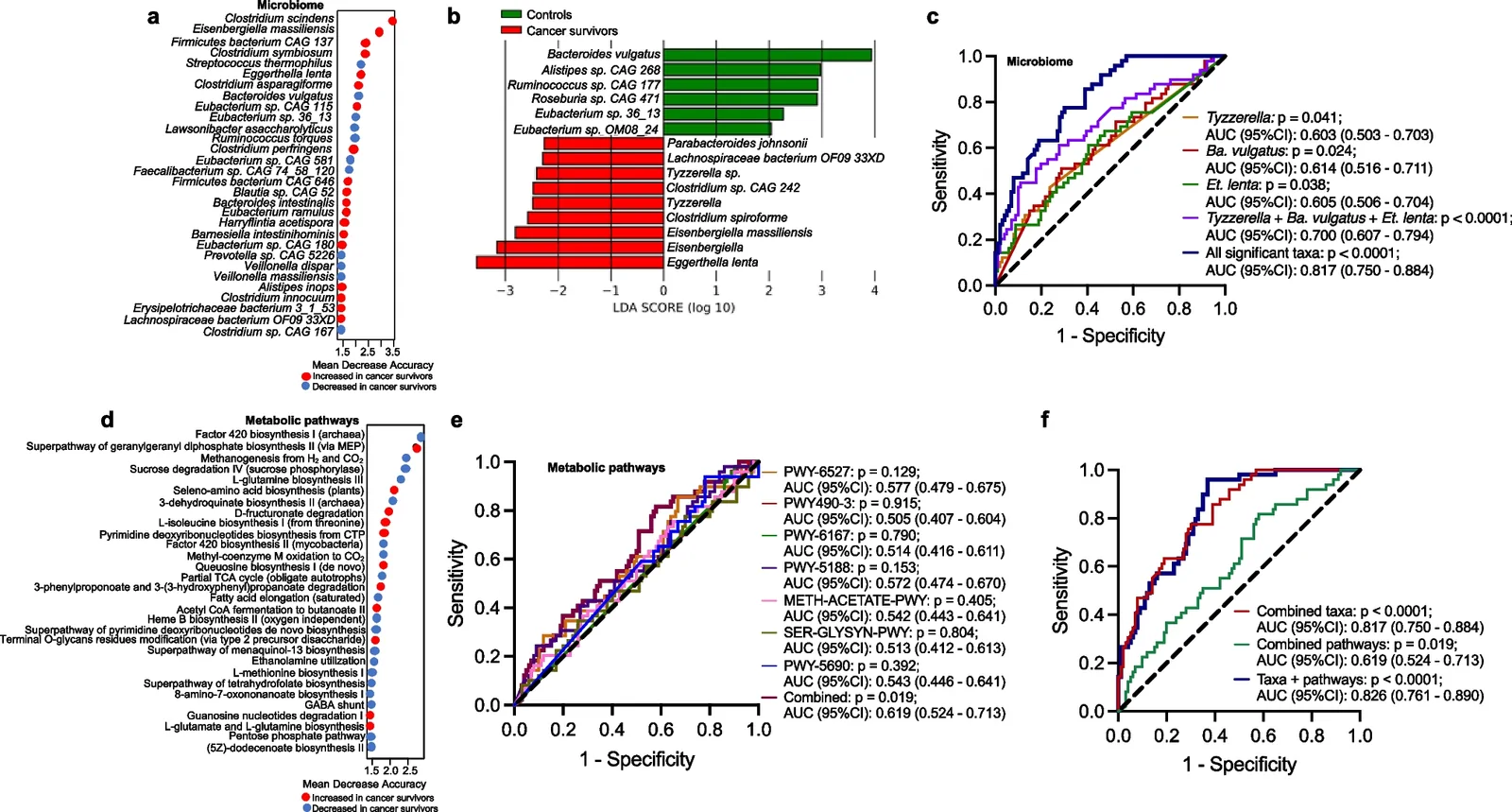
Microbiome signatures and metabolic pathways significantly predict cancer survivorship in older adults. **a** RFA showing the top 30 bacterial species for predicting cancer survivorship in our cohort. **b** LefSe analysis showing the top bacterial taxa that were determined as biomarkers for the gut of cancer survivors (red) and non-cancer controls (green). **c** ROC curves and calculated AUC (with 95% confidence intervals) for the diagnostic ability of the significantly different taxa in the cancer survivor group compared to their controls. The “Combined” curve shows the prediction and AUC for all bacterial and viral taxa that were statistically different in the cancer survivor gut and fit the multiple logistic regression model. **d** RFA showing the top 30 predictive metabolic pathways in the two groups. **e** ROC analyses for predicted metabolic pathways. The “Combined” curve shows the prediction and AUC for all metabolic pathways that were statistically different in the cancer survivor gut and fit the multiple logistic regression model. **f** ROC analyses for taxa, metabolic pathways, and the combination of both. Abbreviations: AUC, area under the curve; *Ba. vulgatus*, *Bacteroides vulgatus*; CTP, cytidine triphosphate; *Et. lenta*, *Eggerthella lenta*; GABA, gamma-aminobutyric acid; LefSe, linear discriminant analysis effect size; MEP, methylerythritol 4-phosphate; RFA, random forest analysis; ROC, receiver operating characteristic; TCA, tricarboxylic acid; 95%CI, 95% confidence interval

After elucidating that the gut microbiome of cancer survivors is compositionally and functionally distinct, we sought to determine if specific microbial taxa or metabolic pathways could differentiate cancer survivors from non-cancer controls. The RFA shows the top 30 bacterial species which have the highest importance for differentiating the cancer survivor gut (Fig. 2a). *Clostridium (Cs.) scindens*, *Eisenbergiella massilensis*, and *Firmicutes (Fm.) bacterium* CAG 137 had the highest mean decrease accuracy, suggesting they have the highest importance for differentiating cancer survivors from controls. Additionally, *Et. lenta*, *Ba. vulgatus*, and *Ls. bacterium* OF09 33XD also showed significant predictive ability in the RFA, and they were found to have significantly different abundances in cancer survivors compared to controls (Fig. 1f). LefSe analysis also shows that *Et. lenta* was the most prominent biomarker for the cancer survivor gut, while *Ba. vulgatus* was the most prominent for non-cancer controls (Fig. 2b). Intriguingly, other taxa that we found to be significantly increased or decreased in the cancer survivor gut, including *Tyzzerella*, *Ls. bacterium* OF09 33XD, and *Eubacterium* (*Eu.*) sp. OM08_24 were also significant biomarkers according to the LefSe. Furthermore, ROC curves and AUC analysis (based on multiple regression analysis) demonstrated that *Tyzzerella* (AUC 0.603 [95%CI 0.503–0.703]; *p* = 0.0412), *Ba. vulgatus* (AUC 0.614 [95%CI 0.516–0.711]; *p* = 0.0243), and *Et. lenta* (AUC 0.605 [95%CI 0.506–0.704]; *p* = 0.0377) significantly predicted cancer survivorship (Fig. 2c). Interestingly, the combination of these three taxa (AUC 0.700 [95%CI 0.607–0.794]; *p* < 0.0001) along with the combination of other significant bacterial and viral taxa (Fig. 1f, Supplementary Figure S3f) significantly increased the prediction (AUC 0.817 [95%CI 0.750–0.884]; *p* < 0.0001). Overall, these data suggest that certain taxa, specifically bacteria, have the potential to be biomarkers for identifying cancer survivorship in older adults.

Furthermore, our RFA analysis for predicted microbial metabolic pathways shows that (1) factor 420 biosynthesis I (archaea), (2) superpathway of geranylgeranyl diphosphate biosynthesis II (via MEP [methylerythritol 4-phosphate]), and (3) methanogenesis from H_2_ and CO_2_ had the most significance in identifying cancer survivorship (Fig. 2d). Interestingly, none of the pathways that were up- or downregulated in the cancer survivor gut compared to controls were in the top 30 predictors on the RFA, suggesting that metabolic pathways may not identify cancer survivorship as strongly, compared to the microbial taxa. This speculation is further supported by our ROC analysis, which shows that none of the up- or downregulated pathways (Fig. 1h) were significant predictors of cancer survivorship (Fig. 2e). However, combining all significantly different pathways together improved the predictive ability (AUC 0.619 [95%CI 0.524–0.713]; *p* = 0.019). Interestingly, when comparing the differentiating capacity of the microbiome to that of the pathways, we found that the microbiome was much higher (AUC 0.817 [95%CI 0.750–0.884]; *p* < 0.0001), even more so than the microbiome and pathways together (Fig. 2f). Thus, these data collectively support that distinct microbiome taxonomic signatures have stronger prediction ability than metabolic pathways for differentiating cancer survivorship in older adults.

### Unique microbiome signatures are linked with cognitive impairment (CI) in older cancer survivors compared to non-cancer controls

**Fig. 3:**
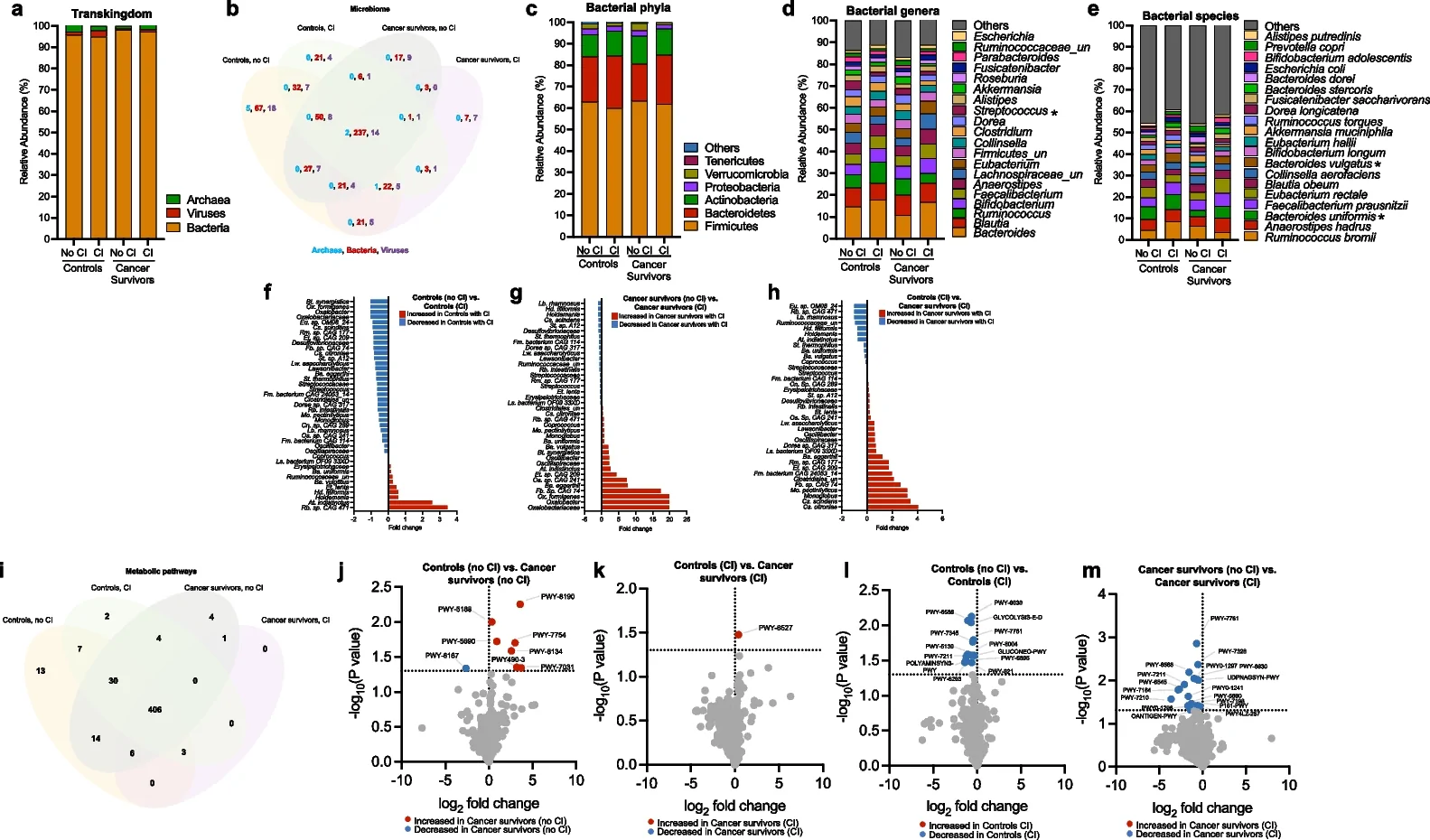
Distinct microbiome compositions and functions in the gut of older adult cancer survivors are linked with cognitive function. **a** Transkingdom abundances of bacteria, viruses, and archaea in the four groups. **b** Venn diagram showing the unique and common archaea, bacteria, and viruses among the four groups. **c** Relative abundances of major bacterial phyla. **d** Relative abundances of major bacterial genera. **e** Relative abundances of major bacterial species. **f–h** Bar charts showing the increased (red) and decreased (blue) taxonomy at the family, genus, and species levels for the following comparisons: **f** controls (no CI) versus controls (CI), **g** cancer survivors (no CI) versus cancer survivors (CI), and **h** controls (CI) versus cancer survivors (CI), represented as fold change values. **i** Venn diagram showing unique and shared predicted metabolic pathways in the four groups. **j–m** Volcano plots showing the increased (red) and decreased (blue) metabolic pathways for **j** controls (no CI) versus cancer survivors (no CI), **k** controls (CI) versus cancer survivors (CI), **l** controls (no CI) versus controls (CI), and **m** cancer survivors (no CI) versus cancer survivors (CI). Volcano plots show the difference in the log_2_ fold change and values less than -log_10_(0.05) were considered statistically significant. Abbreviations*: At. indistinctus*, *Alistipes indistinctus*; *Ba. eggerthii*, *Bacteroides eggerthii*; *Ba. uniformis*, *Bacteroides uniformis*; *Ba. vulgatus*, *Bacteroides vulgatus*; *Bt. synergistica*, *Butyricimonas synergistica*; CI, cognitive impairment; *Cn.* sp. CAG 289, *Collinsella* sp. CAG 289; *Cs. citroniae*, *Clostridium citroniae*; *Cs. scindens*, *Clostridium scindens*; *Et. lenta*, *Eggerthella lenta*; *Et.* sp. CAG 209, *Eggerthella* sp. CAG 209; *Eu.* sp. OM08_24, *Eubacterium* sp. OM08_24; *Fb.* sp. CAG 74, *Faecalibacterium* sp. CAG 74; *Fm. bacterium* CAG 114, *Firmicutes bacterium* CAG 114; *Fm. bacterium* CAG 24053_14, *Firmicutes bacterium* CAG 24053_14; *Hd. filiformis*, *Holdemania filiformis*; *Lb. rhamnosus*, *Lactobacillus rhamnosus*; *Ls. bacterium* OF09 33XD, *Lachnospiraceae bacterium* OF09 33XD; *Lw. asaccharolyticus*, *Lawsonibacter asaccharolyticus*; *Mo. pectinilyticus*, *Monoglobus pectinilyticus*; *Os.* sp. CAG 241, *Oscillibacter* sp. CAG 241; *Ox. formigenes*, *Oxalobacter formigenes*; *Rb. intestinalis*, *Roseburia intestinalis*; *Rb.* sp. CAG 471, *Roseburia* sp. CAG 471; *Rm.* sp. CAG 177, *Ruminococcus* sp. CAG 177; *St.* sp. A12, *Streptococcus* sp. A12; *St. thermophilus*, *Streptococcus thermophilus*. The values with *p < 0.05, **p < 0.01, and ****p < 0.0001 are statistically significant using unpaired *t*-tests (with Welch’s correction, as appropriate, for unequal variances)

Emerging evidence suggests that abnormalities in the gut microbiome contribute to MCI, dementia, and other cognitive dysfunctions in older adults [10, 31]. However, it remains unknown if the gut microbiome is distinctly linked with CI in cancer survivors compared to non-cancer older adults. Interestingly, although the MoCA scores were significantly lower in both cohorts with CI, regardless of cancer history, there was a higher proportion of older adults with CI (40.82%) in the cancer survivor cohort compared to the non-cancer control cohort (36.63%), but the difference was not statistically significant (Supplementary Figure S5a,b). We did not detect any differences in the transkingdom abundances of bacteria, viruses, or archaea among the groups, but there were 67, 21, 17, and 7 unique bacterial species and 18, 4, 9, and 7 unique virus species in controls without CI, controls with CI, cancer survivors without CI, and cancer survivors with CI, respectively (Fig. 3a, b). The β-diversity and α-diversity indices (i.e., Shannon index, Simpson index, and Chao1) were not different among the groups at the transkingdom or bacterial levels (Supplementary Figure S5c-f, h-j). However, the number of observed OTUs was significantly lower in controls with CI, compared to their cognitively healthy counterparts, at both the transkingdom and bacterial levels (Supplementary Figure S5g,k), suggesting that CI may be linked with lower α-diversity.

Next, we show the relative abundances of Gram-positive and Gram-negative bacteria in the four groups (Supplementary Figure S6a). The abundances of Gram-negative taxa were enriched in non-cancer controls with CI (34.69%) and cancer survivors with CI (32.49%), compared to their cognitively healthy counterparts, but the differences were not significant. Furthermore, phylogenic analyses revealed no significant differences in the major bacterial phyla or in the F/B ratio (Fig. 3c; Supplementary Figure S6b). However, the abundance of family *Streptococcaceae* was significantly lower in the gut of non-cancer controls with CI compared to their non-CI counterparts (Supplementary Figure S6c, d). Furthermore, there were five other bacterial families and eight bacterial genera which were significantly different among cancer survivors and their controls with and without CI (Fig. 3d, Supplementary Figure S6e-q). Interestingly, we observed that the abundances of *Ba. uniformis* and *Ba. vulgatus* were decreased in cancer survivors without CI, compared to their non-cancer without CI controls, while their abundance increased in both cancer survivors and controls with CI (Fig. 3e, Supplementary Figure S7a, b). This suggests that the changes in these bacterial species were specific to cognitive function and independent of cancer survivorship. Additionally, we observed that the abundances of *Butyricimonas synergistica*, *Oxalobacter (Ox.) formigenes*, *Oxalobacter*, and *Oxalobacteriacae* were the most decreased, while the abundances of *Roseburia* (*Rb.*) sp. CAG 471 and *Alistipes (At*.) *indistinctus* were most increased in non-cancer older adults with CI compared to non-CI controls (Fig. 3f). The abundances of *Lactobacillus* (*Lb.*) *rhamnosus* and *Holdemania filiformis* were the most decreased taxa in cancer survivors with CI compared to non-CI cancer survivors, while *Oxalobacteriaceae*, *Oxalobacter*, and *Ox. formigenes* were the most increased taxa (Fig. 3g). Similarly, the abundances of *Eu.* sp. OM08_24, *Rb.* sp. CAG 471, and *Lb. rhamnosus* were the most decreased, while the abundances of *Cs. citroniae*, *Cs. scindens*, and *Monoglobus* were most increased in older cancer survivors with CI compared to non-cancer controls with CI (Fig. 3h; Supplementary Figure S7c-t; S8a-g). These results indicate that older cancer survivors harbor unique gut microbiome signatures that are linked with CI and are distinct from those of older adults with CI and no history of cancer.

Furthermore, we observed no significant differences in α-diversity of viruses (Supplementary Figure S9a-c) or in the abundances of major virus families (Supplementary Figure S9d) among all four groups of older adults with and without CI and/or history of cancer and their controls. However, at the species level, the abundance of *Lb. phage* A2 was significantly depleted in the gut of cancer survivors with CI compared to their non-cancer controls with CI, while *Lactococcus phage* bIL312 was significantly lower in non-cancer controls with CI compared to their cognitively healthy counterparts (Supplementary Figure S9e-g). The relative abundances of the major archaea species were not significantly distinct among the groups (Supplementary Figure S8h). Collectively, our data indicate that bacterial and viral signatures are unique to older cancer survivors with or without CI. However, the precise mechanisms of these bacteria and viruses and their contributions to cancer treatment-related side effects and poor gut and brain health in cancer survivors remain elusive.

### Metabolic functions of the microbiome are distinctly linked among cancer survivors with CI and without compared to their respective controls

Next, we sought to determine if distinct metabolic functions are linked with CI, cancer survivorship, or both. Our PCA shows no significant differences in the clustering of the metabolic pathways for the four groups (Supplementary Figure S10). Interestingly, we found 13, 2, and 4 pathways unique to the non-cancer healthy control, non-cancer with CI, and cancer non-CI control groups, respectively (Fig. 3i; Supplementary Table S4). Furthermore, the abundances of metabolic pathways related to L-glutamate degradation XI (PWY-8190), bile acid 7- and α-dehydroxylation (PWY-7754), bile acid 7- and β-dehydroxylation (PWY-8134), protein N-glycosylation by bacteria (PWY-7031), assimilatory nitrate reduction VI (PWY490-3), tetrapyrrole biosynthesis I from glutamate (PWY-5188), and TCA cycle (plants and fungi, PWY-5690) were significantly increased, while flavin biosynthesis by archaea (PWY-6167) was significantly decreased in cognitively healthy cancer survivors compared to cognitively healthy controls (Fig. 3j). This suggests that microbial metabolic pathways differ in our cancer survivor cohort, independent of CI. Stachyose degradation (PWY-6527) was the only pathway to be significantly increased in cancer survivors with CI compared to non-controls with CI (Fig. 3k), while 13 metabolic pathways were significantly decreased in the gut of non-cancer controls with CI, compared to non-cancer controls without CI (Fig. 3l). Similarly, 17 pathways were significantly decreased in the gut of cancer survivors with CI compared to their no CI cancer survivor controls (Fig. 3m). Supplementary Table S5 shows all of the increased and decreased metabolic pathway names, their descriptions, and log_2_ fold change values for the four groups. Pathways related to nucleotide metabolism and tyrosine biosynthesis were most significantly reduced in the gut of older adults with CI compared to their controls irrespective of their cancer history, suggesting that the depletion of metabolites of these pathways may have roles in CI pathology in older adults, independent of cancer history. These data provide evidence that functional changes in the gut microbiome distinctly occur based on cancer history and/or prevalence of CI.

### Microbiome signatures and metabolic pathways can differentiate those with CI from those of cognitively healthy older cancer survivors

**Fig. 4:**
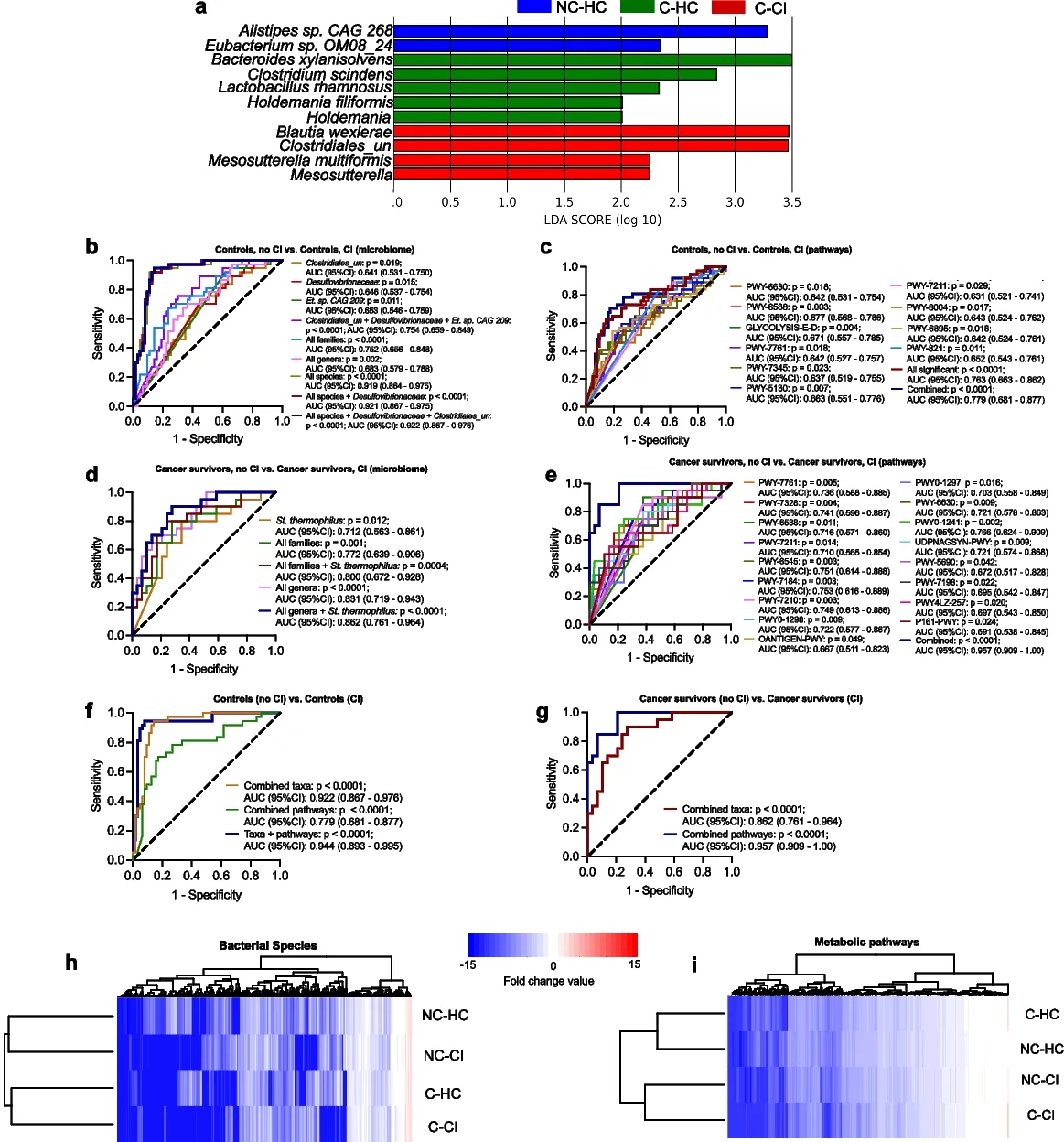
Gut microbiome signatures and their functions can predict cancer survivorship and cognitive function in older adults. **a** LefSe analysis showing the bacterial taxa and biomarkers increased in non-cancer controls without CI (blue), cancer survivors without CI (green), and cancer survivors with CI (red). **b**,** c** ROC curves showing the diagnostic ability of **b** bacterial and viral taxa and **c** metabolic pathways significantly changed in non-cancer controls with CI compared to those who were cognitively healthy. The “All significant combined” curve in **c** shows the diagnostic ability and AUC for all significant pathways (according to logistic regression), while “All combined” includes all pathways that fit the logistic model, regardless of if they were significant or not on the regression analysis. **d**,** e** ROC curves showing the diagnostic ability of **d** bacterial and viral taxa and **e** metabolic pathways significantly changed in cancer survivors with CI compared to survivors without CI. The “Combined” curve in **e** shows the effect of all metabolic pathways statistically different in cancer survivors with CI compared to their cognitively healthy counterparts. **f**,** g** ROC curves comparing the diagnostic ability of the microbiome, pathways, and/or the combination of both for **f** non-cancer controls with CI versus cognitively healthy controls and **g** cancer survivors with CI versus cognitively healthy cancer survivors. **h**,** i** Clustered heatmaps for the four groups (NC-HC, NC-CI, C-HC, and C-CI) based on **h** bacterial species and **i** metabolic pathways. The heatmaps are shaded based on fold change values. Abbreviations: AUC, area under the curve; C-CI, cancer survivors with cognitive impairment group; C-HC, cancer survivors without cognitive impairment group; CI, cognitive impairment; *Et.* sp. CAG 209, *Eggerthella* sp. CAG 209; LefSe, linear discriminant analysis effect size; NC-CI, non-cancer controls with cognitive impairment group; NC-HC, non-cancer controls without cognitive impairment group; ROC, receiver operating characteristic; *St. thermophilus*, *Streptococcus thermophilus*; 95%CI, 95% confidence interval

Our next goal was to determine if gut microbiome signatures can predict CI in older adults with and without cancer history. LefSe analysis showed that enrichment of *At.* sp. CAG 268 and *Eu.* sp. OM08_24 are potential biomarkers for healthy cognition in non-cancer older adults, while increased abundances of *Ba. xylansolvens* and *Cs. scindens* are indicators of healthy cognition in cancer survivors (Fig. 4a). Furthermore, enriched abundances of *Blautia wexlerae* and unclassified *Clostridiales* are potential biomarkers for CI in cancer survivors (Fig. 4a). Our ROC and AUC analyses also suggest that unclassified *Clostridiales*, *Desulfovibrionaceae*, and *Et.* sp. CAG 209 individually could predict CI in controls, but the strongest predictor was all species combined with unclassified *Clostridiales* and *Desulfovibrionaceae* (AUC 0.922 [95%CI 0.867–0.976]; *p* < 0.0001) (Fig. 4b). Similarly, the combination of all metabolic pathways together created the ROC with the highest predictive ability (AUC 0.778 [95%CI 0.681–0.877]; *p* < 0.0001), compared to the pathways individually (Fig. 4c). Interestingly, even a single taxon—*Streptococcus* (*St.*) *thermophilus*—can predict CI in cancer survivors with approximately 71% confidence (AUC 0.712 [95%CI 0.563–0.861]; *p* = 0.0123), but when combined with other significantly changed bacterial genera, its power increased to about 85% (AUC 0.862 [95%CI 0.761–0.964]; *p* < 0.0001) (Fig. 4d). Additionally, our data show that metabolic pathways had a higher potential to predict CI in cancer survivors, with an AUC of 0.957 (95%CI 0.909–1.00) or 95% confidence (*p* < 0.0001; Fig. 4e). We also show that microbiome signatures more strongly predict CI in non-cancer older adults (Fig. 4f), while metabolic pathways show a higher potential to predict CI in older cancer survivors (Fig. 4g).

We also controlled cognitive function to determine the diagnostic ability for cancer survivorship and found that the gut microbiome composition more strongly predicts cancer survivorship in older adults with both healthy cognition and those with CI (Supplementary Figure S11a-d). Additionally, we performed hierarchical clustering to observe patterns in the abundances of bacterial taxa and metabolic pathways among the groups. Intriguingly, bacterial species seemed to cluster based on cancer survivorship (Fig. 4h), while bacterial phyla and genera clustered based on cognitive function (Supplementary Figure S11e,f). Metabolic pathways also clustered based on CI (Fig. 4i), suggesting that metabolic pathways as well as bacterial phyla and genera are able to more precisely differentiate cognitive function in this cohort compared to bacterial species. Collectively, these findings indicate that both the microbiome and its metabolic pathways can strongly differentiate or predict CI and cancer survivorship in older adults.

### Mechanistic understanding of microbiome and metabolic functions with cognitive function and cancer survivorship in older adults

**Fig. 5:**
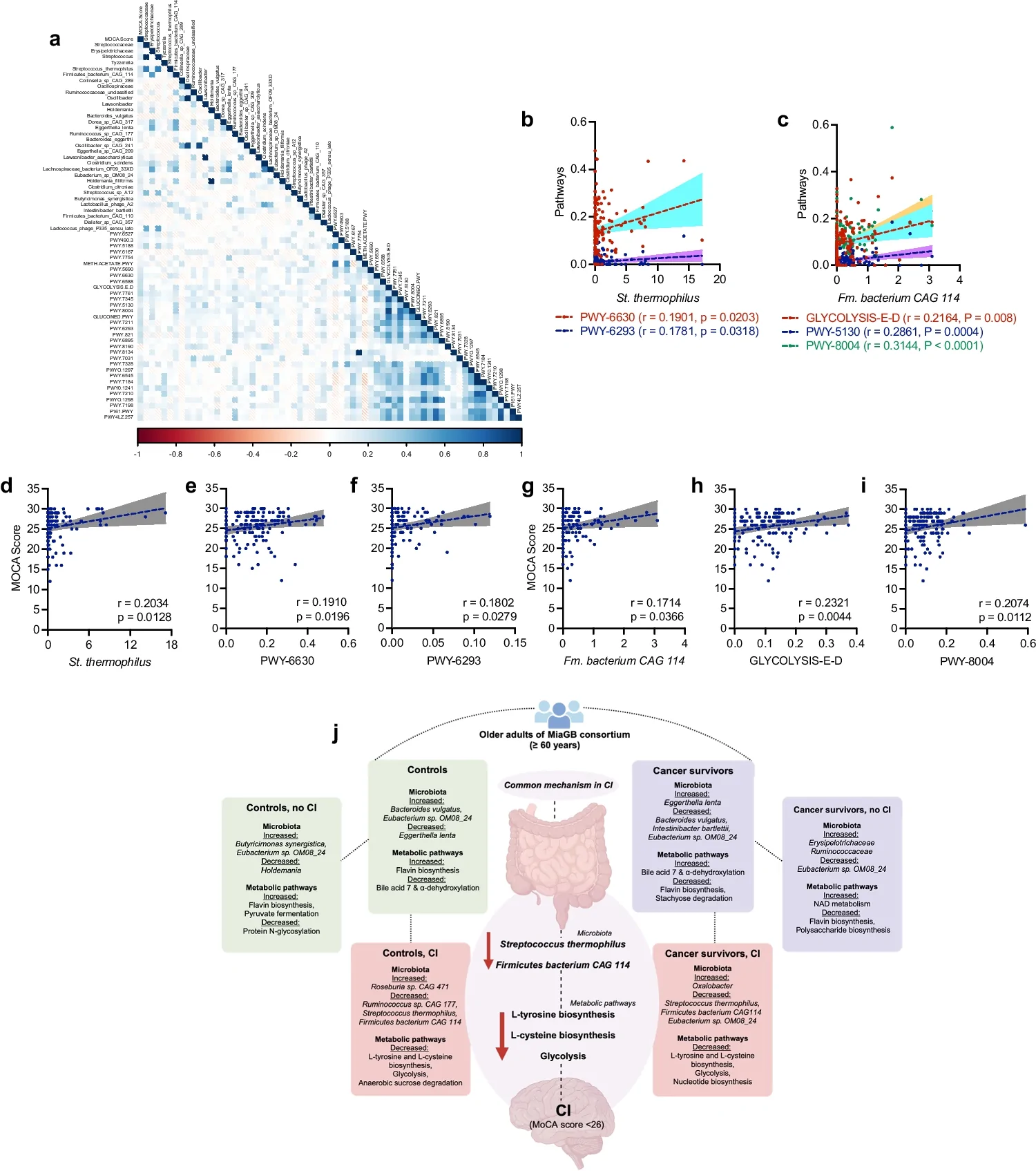
There are significant correlations between unique gut microbiota and metabolic pathways in cancer survivors, and they are linked with cognitive function. **a** Corrplot showing correlations between bacteria/viruses and metabolic pathways and the MoCA score. **b, c** Plots showing significant and positive correlations between specific taxa and metabolic pathways for **b** *St. thermophilus* and **c** *Fm. bacterium* CAG 114, which were the only two taxa significantly correlated with MoCA. **d–i** Individual plots showing correlations between specific bacterial taxa or their metabolic pathways and MoCA scores for **d** *St. thermophilus*, **e** PWY-6630, **f** PWY-6293, **g** *Fm. bacterium* CAG 114, **h** GLYCOLYSIS-E-D, and **i** PWY-8004. **j** Purported model showing that the composition and function of the microbiome in the gut of cancer survivors are distinct, compared to non-cancer older adults. Additionally, gut microbiome composition and function were unique based on cognitive function. Interestingly, we found a common pattern in older adults with CI, irrespective of cancer history. We observed that depletion of beneficial bacteria like *St. thermophilus* and *Fm. bacterium* CAG 114 was linked with decreases in tyrosine and cysteine biosynthesis and glycolysis, which were associated with a decline in cognitive function in older adults with and without a history of cancer. Abbreviations: CI, cognitive impairment; *Fm. bacterium* CAG 114, *Firmicutes bacterium* CAG 114; MiaGB, Microbiome in aging-Gut and Brain; MoCA, Montreal Cognitive Assessment; *St. thermophilus*, *Streptococcus thermophilus*. Pearson’s correlation coefficient was calculated for all correlation analyses. Individual correlation plots show the linear regression line and 95% confidence intervals (shaded regions)

We performed hierarchal correlation clustering to further develop our understanding of whether and how interactions between microbiome signatures and their metabolic pathways are linked with changes in cognitive function in older adults with and without a history of cancer. From our Pearson correlation analyses of all significantly differing taxa and metabolic pathways (Fig. 5a; Supplementary Figure S12), we selected taxa which were positively correlated with specific metabolic pathways, suggesting that these microbes may be majorly carrying out those metabolic pathways (Fig. 5b,c; Supplementary Table S6). We found that two taxa, *St. thermophilus* and *Fm. bacterium* CAG 114, and their respective pathways, including PWY-6630 (L-tyrosine biosynthesis) and PWY-6293 (L-cysteine biosynthesis) (both correlated with *St. thermophilus)*, and GLYCOLYSIS-E-D (superpathway of glycolysis and the Entner-Duodoroff pathway) and PWY-8004 (Entner-Duodoroff pathway) had strong and positive correlations with MoCA (Fig. 5d–i). This indicates that reduced abundance of *St. thermophilus* and *Fm. bacterium* CAG 114 is functionally linked with decreased tyrosine and cysteine biosynthesis and decreased glycolysis, respectively, which are further associated with reduced cognitive function in older adults. We also observed that the abundance of Gram-negative bacteria was slightly increased in the gut of older adults with CI, independent of cancer survivorship. This further suggests that functional and compositional changes in the microbiome are more connected with CI in older adults than cancer survivorship.

Based on these results, we purported a model indicating that the composition and function of the microbiome in the gut of cancer survivors are distinct, characterized by enrichment of *Et. lenta* and bile acid metabolism and decreased abundances of *Ba. vulgatus*, *Intestinibacter bartletti*, and *Eu.* sp. OM08_24, as well as reduced stachyose degradation, compared to non-cancer older adults. Additionally, the gut of cancer survivors with CI was significantly enriched with *Oxalobacter* and depleted of *Eu.* sp. OM08_24, with reduced nucleotide biosynthesis, while the gut of non-cancer controls with CI was highly abundant with *Rb.* sp. CAG 471, with reduced levels of *Rm.* sp. CAG 177 and lower anaerobic sucrose degradation. Interestingly, we found a common pattern in older adults with CI, irrespective of cancer history. We observed that depletion of beneficial bacteria like *St. thermophilus* and *Fm. bacterium* CAG 114 was linked with decreases in tyrosine and cysteine biosynthesis and glycolysis and was associated with a decline in cognitive function in older adults with and without a history of cancer (Fig. 5j).

## Discussion

In this pilot study, we used data from our multi-site MiaGB consortium to address some critical knowledge gaps, including (1) the potential of microbiome signatures to differentiate cancer survivorship and CI in older adults and (2) how changes in the microbiome may be linked with or contribute to CI in older cancer survivors. Interestingly, we found that the gut microbiome of cancer survivors may be structurally and functionally distinct, compared to non-cancer survivor controls. These specific microbiome signatures and their metabolic pathways could serve as potential biomarkers to differentiate cancer survivors among older adults. Furthermore, we show that the microbiome and its metabolic functions are significantly distinct in older cancer survivors experiencing CI compared to their non-cancer controls. In addition, we developed a mechanistic model by which the microbiome and its metabolic pathways may be linked with CI in both cancer survivors and their non-cancer controls.

Emerging evidence suggests that the gut microbiome is significantly impacted by cancer treatments and contributes to cancer progression and cognitive function [20, 32, 33]. Multiple recent studies in animal models show that cancer treatments like chemotherapy (i.e., 5-fluorouracil, methotrexate, irinotecan, and oxaliplatin) significantly change the gut microbiome by reducing microbiota diversity; depleting beneficial microbes like *Lactobacillus*, *Bifidobacterium*, and *Prevotella*; and enriching potential pathogens like Proteobacteria and *Escherichia* [34–38]. Furthermore, such changes are linked with impairments in host fatty acid and energy metabolism, increased intestinal permeability, and activated immune and inflammatory responses [34, 38–40]. A few human studies have demonstrated that combination chemotherapy or conditioning in survivors of various cancer types decreased α-diversity and enriched Proteobacteria, *Staphylococcus*, and *Escherichia coli* while decreasing the abundances of *Bacteroides*, *Ruminococcus*, *Roseburia*, *Lactobacillus*, and *Bifidobacterium* [18, 41, 42]. However, it remains unknown if these changes are short-term or also persist in the long term. The majority of research on cancer and the microbiome focuses on how the microbiome impacts the efficacy of cancer treatments [43], with less known about how cancer treatments affect the human gut microbiota in the long term. Our study addresses this knowledge gap by highlighting the long-term impairments in the microbiome caused by cancer treatments. Here, in line with a previous study [41], we demonstrated that the abundances of *Bacteroides* species, including *Ba. vulgatus*, *Ba. uniformis*, and *Ba. eggerthii*, were reduced in cancer survivors who had been exposed to cancer treatments at least 5 years prior. Interestingly, we also found that distinct microbiome signatures can significantly differentiate older cancer survivors from their non-cancer controls, suggesting the potential development of microbiome-based biomarkers for defining cancer survivorship and assessing health status.

Cancer survivors experience a high burden of comorbidities, including CI, and the microbiome significantly impacts the gut-brain axis and may contribute to the development of CI in older adults [13]. Multiple studies, including ours, show that the gut of older adults with MCI, Alzheimer’s disease, or dementia harbors a significantly distinct microbiome compared to the gut of cognitively healthy controls [10, 15, 44, 45]. Importantly, some emerging studies report that depletion of beneficial microbes in the gut of animal models or patients with CI and/or dementia is linked with increased inflammation and reduced production of beneficial metabolites [45, 46]. However, changes in the microbiome of older cancer survivors with and without CI and how they contribute to cognitive health have not been studied. We observed that species of *Lactobacillus* (*Lb. rhamnosus*), *Roseburia* (*Rb. intestinalis*), and *Ruminococcus* (*Rm.* sp. CAG 177) were significantly depleted in cancer survivors with CI, while *Ba. vulgatus*, *Ba. uniformis*, and *Ba. eggerthii* were enriched, compared to cognitively healthy controls. *Lb. rhamnosus*, *Rb. intestinalis*, and *Rm.* sp. CAG 177 are considered beneficial bacteria because they produce metabolites like short-chain fatty acids, which have positive effects on many host cells, including colonocytes and neurons, and have been shown to improve cognitive function and brain health [47–49]. These results indicate that the gut of cancer survivors experiencing CI harbors a microbiome lacking beneficial bacteria, which may be contributing to cognitive decline.

Although the precise mechanisms are unknown, previous studies reported that gut microbiota dysbiosis induced by cancer treatments increased circulating matrix metalloproteinases and pro-inflammatory markers (i.e., tumor necrosis factor and interleukin-1β) [41] and modulated multiple metabolic processes including energy metabolism, nucleotide biosynthesis and degradation, signal transduction pathways, metabolism of cofactors, vitamins, and glycans, and degradation of xenobiotics [18]. Interestingly, our prediction analyses also revealed that cancer survivors with CI had reduced nucleotide metabolism, including de novo biosynthesis and degradation of pyrimidines. Additionally, inflammation is a central mechanism underlying the development of CI or other brain dysfunctions and is increased in gut dysbiosis and with the use of cancer treatments [41, 50–52]. Although no inflammatory pathways were significantly altered in our cohorts, potential inflammatory bacteria including *Tyzzerella*, *Et. lenta*, and *Ls. bacterium* OF09 33XD were enriched in the gut of cancer survivors, irrespective of cognitive function. *Tyzzerella* species have been identified as opportunistic pathogens, and their enrichment has been linked with inflammation [53], and in patients with Crohn’s disease [54], cardiovascular diseases [55], or increased fat mass [56]. Furthermore, *Et. lenta* has been reported with gut dysbiosis and inflammation in rheumatoid arthritis [57], bacteremia [58, 59], and colitis [60]. Multiple *Lachnospiraceae* are butyrate-producers and, thus, may have anti-inflammatory effects or beneficial impacts on intestinal health [61]; however, this family has also been implicated with inflammation [62]. Enrichment of Gram-negative bacteria may contribute to inflammation due to its high deposition of lipopolysaccharide and other inflammatory cellular ingredients [63]. We did observe an increase in Gram-negative taxa in older adults with CI, irrespective of cancer history, but the differences were not statistically significant. On the other hand, anti-inflammatory and well-known probiotic species, including *Lb. rhamnosus* and *St. thermophilus*, were depleted in the gut of cancer survivors with CI, suggesting that inflammatory characteristics may contribute to the development of CI, at least in part. However, additional studies like ours are warranted to uncover the role of inflammatory pathways and others in cancer treatment-induced gut dysbiosis and CI.

For further mechanistic understanding, we performed hierarchical correlation clustering, which revealed that *St. thermophilus* was positively correlated with metabolic pathways related to the biosynthesis of amino acids—L-cysteine and L-tyrosine. Cysteine and tyrosine are both non-essential amino acids, meaning they are synthesized in our body, and have known roles in human metabolism and cognitive function. L-cysteine and its precursor, N-acetylcysteine (NAC), are also precursors of glutathione, which is a potent antioxidant with purported neuroprotective effects. NAC treatment in humans and mice has been associated with improvements in cognitive function and improved metabolism in the brain via reductions in oxidative stress biomarkers and anti-inflammation [64–66]. Tyrosine is a precursor for catecholamine neurotransmitters (i.e., dopamine), and its supplementation has also been linked with improved cognitive function [67]. Thus, our findings suggest that depletion of *St. thermophilus* was linked with increased CI, especially in cancer survivors, and this could be due to reduced biosynthesis of L-cysteine and L-tyrosine. Additionally, *Fm. bacterium* CAG 114 was significantly correlated with CI and could be a biomarker for predicting CI in non-cancer older adults. Our hierarchal clustering showed that this bacterial species was correlated with pathways related to energy metabolism, particularly Entner-Duodoroff glycolysis. Impaired or reduced glycolysis results in less energy production, which has detrimental impacts on metabolism and can severely damage the brain, leading to the development of CI, dementia, or Alzheimer’s disease [68]. Thus, our findings suggest that depletion of *Fm. bacterium* CAG 114 in the gut of non-cancer survivor controls with CI (compared to their cognitively healthy counterparts) was linked with decreased energy production via glycolysis. However, more studies in preclinical models are warranted to better elucidate the mechanisms of both *St. thermophilus* and *Fm. bacterium CAG 114* in the context of cognitive function.

To the best of our knowledge, this is the first study to employ metagenomics to profile the gut microbiota structure and function of cancer survivors, link it with cognitive function, and predict the potential of the composition and function of the gut microbiota to serve as a biomarker for cancer survivorship and/or CI in older adults. One strength of our metagenomics approach is that we could identify the viral and archaeal components of the microbiota, while 16 s rRNA sequencing only allows for identification of bacterial taxa [69]. Our recent study suggests that older adults with CI have significantly different viromes and abundances of bacteriophages compared to cognitively healthy controls [26]. Here, we found that a few bacteriophages, including *Lb. phage* A2 and *Lactococcus phage P335 *sensu lato, were significantly altered in cancer survivors with and without CI. Although enrichment of these phages was linked with pathways related to bile acid metabolism and others, there were no significant correlations between these phages and potential bacterial hosts. Therefore, future studies are warranted to characterize the viral components of the gut microbiota (including the phageome) in older adult cancer survivors, elucidate the bacteria-phage interactions, and link them with cognitive health. Additionally, although one previous study quantified the abundances of major archaea in patients with colorectal cancer [70], there are no known studies regarding changes in the virome or archaeome in cancer survivors and with consideration to cognitive function. We found no significant differences in major archaeal species in any of our cohorts. Further studies are necessary to confirm these findings.

This study has some limitations that should be addressed. First, our metagenomics analyses only allow us to predict metabolic pathways and do not reflect actual bacterial metabolic activities. Thus, to better elucidate bacterial metabolism in cancer survivors and its link with cognitive function, future studies should combine untargeted and targeted metabolomic analyses to report bacterial metabolites in the serum, plasma, or stool. Another limitation is that the cancer type, treatment type, and treatment duration in our cancer survivor cohorts remain unknown. Our demographic surveys for the MiaGB study do not include questions related to these parameters. However, it is also a strength that despite these factors being unknown, older cancer survivors still demonstrated significantly distinct microbiome signatures compared to non-cancer older adults. Although this does increase the generalizability of our findings, it is important to conduct additional studies in specialized cohorts (i.e., cohorts with a specific kind of cancer) to better understand if microbiome structure and function are unique based on cancer type or treatment type/duration and how these potential differences are linked with cognitive function. This is difficult because cancer treatments are often complex and highly vary depending on cancer type, severity, and are prescribed on a patient-to-patient basis. Nonetheless, previous studies have suggested that these factors, and many others, significantly influence gut microbiota following treatment [71, 72]. Third, our study findings are limited to a single time point and are not representative of changes to microbiome structure and function or cognition over a period of time. The gut microbiome is highly modifiable and likely changes compositionally and functionally over time following cancer treatment, which leads to high variability depending on time since treatment. The Chemo-Gut study assessed microbiome signatures at multiple time points post treatment, reporting differences in diversity and specific taxonomy at < 6 months compared to > 6 months following treatment and highlighting variability in time-since-treatment [20]. Despite these preliminary findings, most studies include a pre-post design, limiting our understanding of the longitudinal effects of cancer treatments on the microbiome [73]. Future studies should include analyses of the microbiome and cognitive function over several time points post-treatment so that we can fully elucidate these changes over time in older adult cancer survivors. Furthermore, our pilot analysis includes a small sample size, especially for our cancer survivor cohort. Future studies with larger sample sizes are necessary to confirm our findings. Additionally, although complex, it is critical to understand if and how specific cancer treatments, cancer types, and treatment duration impact the gut microbiome and brain health in older adult cancer survivors. Such studies will aid in the development of novel and natural microbiome-based therapies to maintain or improve quality of life in cancer survivors. It is important to consider that there were significant differences in the ages of our cancer survivor and cancer survivor with CI cohorts, compared to their controls. Aging is a major risk factor for CI and is also associated with gut microbiota dysbiosis [9, 74]. Therefore, it is possible that aging significantly contributed to the changes observed in the gut microbiota, its predicted metabolic functions, and the development of CI. Future analyses should consider age-matching to determine if specific signatures and cognitive functions are prevalent, regardless of age. Previous studies have shown that the gut microbiome is modified by other factors, including diet [15, 75] and medication use (i.e., antibiotics, proton pump inhibitors, and laxatives) [76]. Our demographic surveys include questions related to diet (vegetarian versus non-vegetarian or yogurt/probiotics consumption), medication use, and other factors like disease history or lifestyle habits such as smoking. Thus, future studies should include univariate or multivariate analyses to minimize the effects of these confounding variables. Such analyses would allow us to better understand how gut microbiome structure and function change due to cancer treatments alone and how this is linked with cognitive function in older adults.

## Supplementary Information

Below is the link to the electronic supplementary material.

ESM 1 (DOCX 17.0 KB)

ESM 2 (PPTX 4.50 MB)

ESM 3 (DOCX 19.1 KB)

ESM 4 (DOCX 16.6 KB)

ESM 5 (DOCX 17.8 KB)

ESM 6 (DOCX 23.2 KB)

ESM 7 (DOCX 24.2 KB)

## Data Availability

Data from the metagenomics sequencing are available at NCBI SRA project no. PRJNA1142892, at the following link: https://dataview.ncbi.nlm.nih.gov/object/PRJNA1142892?reviewer=6gm9v61b1c8u2qq9edj3lnhj78.
