# Supplementary material for: Gut microbiome signatures predict cognitive impairment in older cancer survivors
Source: GeroScience. 2025 Nov 1;48(4):5575–96. doi: 10.1007/s11357-025-01917-x (PMC13575041; doi:10.1007/s11357-025-01917-x)
Supplement: Supplementary file 1 [file 11357_2025_1917_MOESM1_ESM.docx]

| **Demographic** | **All Participants** |  |
| --- | --- | --- |
| Number of participants in cohort | 150 |  |
| Female (%) | 100 (66.67%) |  |
| Male (%) | 50 (33.33%) |  |
| Age (years) | 73.16 ± 0.66 |  |
| BMI (kg/m^2^) | 26.84 ± 0.44 |  |
| Ethnicity (%)  Not Hispanic or Latino  Hispanic or Latino  Unknown ethnicity/unreported | 122 (81.33%)  17 (11.33%)  11 (7.33%) |  |
| Race (%)  White  Black or African American  Asian  Unknown race/unreported | 137 (91.33%)  4 (2.67%)  5 (3.33%)  4 (2.67%) |  |

**Supplementary Table S1.** Participant demographics.

All data are presented as mean ± SEM or frequency (%), as appropriate.

Abbreviations: BMI: Body mass index, SEM: Standard error of the mean
