# Supplementary material for: Gut microbiome signatures predict cognitive impairment in older cancer survivors
Source: GeroScience. 2025 Nov 1;48(4):5575–96. doi: 10.1007/s11357-025-01917-x (PMC13575041; doi:10.1007/s11357-025-01917-x)
Supplement: Supplementary file 2 [file 11357_2025_1917_MOESM3_ESM.docx]

**Supplementary Table S2.** Unique pathways and descriptions in cancer survivors compared to non-cancer controls.

| **Controls (22 unique pathways)** | | |
| --- | --- | --- |
| PWY-3801 | | Sucrose degradation II (sucrose synthase) |
| PWY-6763 | | Salicortin biosynthesis |
| PWY-7165  PWY-7396 | | L-ascorbate biosynthesis VIII (engineered pathway)  Butanol and isobutanol biosynthesis (engineered |
| URSIN-PWY | | Ureide biosynthesis |
| PWY-5654 | | 2-amino-3-carboxymuconate semialdehyde degradation to 2-hydroxypentadienoate |
| P163-PWY | | L-lysine fermentation to acetate and butanoate |
| P162-PWY | | L-glutamate degradation V (via hydroxyglutarate) |
| PWY-7820  DENITRIFICATION-PWY  PWY-6662  P562-PWY  PWY-5101  PWY-5028  PWY-7039  PWY-6748  PWY-7853  PWY-5742  P241-PWY  PWY-7527  PWY-7294  PWY-4361 | | Teichuronic acid biosynthesis (*B. subtilis* 168)  Nitrate reduction I (denitrification)  Superpathway of quinolone and alkylquinolone biosynthesis  Myo-inositol degradation I  L-isoleucine biosynthesis II  L-histidine degradation II  Phosphatidate metabolism, as a signaling molecule  Nitrate reduction VII (denitrification)  6-hydroxymethyl-dihydropterin diphosphate biosynthesis V (*Pyrococcus*)  L-arginine degradation IX (arginine:pyruvate transaminase pathway)  Coenzyme B biosynthesis  L-methionine salvage cycle III  D-xylose degradation IV  S-methyl-5-thio-α-D-ribose 1-phosphate degradation I |
| **Cancer survivors (5 unique pathways)** | | |
| UDPNACETYLGALSYN-PWY | UDP-N-acetyl-D-glucosamine biosynthesis II | |
| PWY-7279 | Aerobic respiration II (cytochrome c) (yeast) | |
| PWY-6113  PWY-7317 | Superpathway of mycolate biosynthesis  Superpathway of dTDP-glucose-derived O-antigen building blocks biosynthesis | |
| PWY-5724 | Superpathway of atrazine degradation | |

Abbreviations: *B. subtilis*: *Bacillus subtilis*, dTDP: Deoxythymidine diphosphate, UDP: Uridine diphosphate
