# Supplementary material for: Gut microbiome signatures predict cognitive impairment in older cancer survivors
Source: GeroScience. 2025 Nov 1;48(4):5575–96. doi: 10.1007/s11357-025-01917-x (PMC13575041; doi:10.1007/s11357-025-01917-x)
Supplement: Supplementary file 3 [file 11357_2025_1917_MOESM4_ESM.docx]

**Supplementary Table S3.** Significantly increased and decreased metabolic pathways in cancer survivors compared to their controls.

| **Pathway** | | | **Pathway Description** | **log_2_(fold change)** | **-log_10_(p value)** |
| --- | --- | --- | --- | --- | --- |
| PWY-6527 | | Stachyose degradation | | 0.2603 | 2.0333 |
| PWY490-3 | | Nitrate reduction VI (assimilatory) | | 3.6055 | 2.0283 |
| PWY-5188  PWY-6167 | | Tetrapyrrole biosynthesis I (from glutamate)  Flavin biosynthesis II (archaea) | | 0.2715  -2.9110 | 1.7975  1.7270 |
| PWY-7754 | | Bile acid 7 & a-dehydroxylation | | 2.4075 | 1.5649 |
| METH-ACETATE-PWY | | Methanogenesis from acetate | | 0.2988 | 1.4176 |
| PWY-5690 | | TCA Cycle II (plants and fungi) | | 0.6842 | 1.3708 |
| SER-GLYSYN-PWY | | Superpathway of L-serine & glycine biosynthesis I | | 0.2153 | 1.3643 |
|  |  | | |  |  |

Abbreviations: TCA: Tricarboxylic acid
