# Supplementary material for: Gut microbiome signatures predict cognitive impairment in older cancer survivors
Source: GeroScience. 2025 Nov 1;48(4):5575–96. doi: 10.1007/s11357-025-01917-x (PMC13575041; doi:10.1007/s11357-025-01917-x)
Supplement: Supplementary file 4 [file 11357_2025_1917_MOESM5_ESM.docx]

**Supplementary Table S4.** Unique metabolic pathways among the four groups.

| **Controls, no CI (13 unique pathways)** | |
| --- | --- |
| PWY-3801 | Sucrose degradation II (sucrose synthase) |
| PWY-6763 | Salicortin biosynthesis |
| PWY-7396 | Butanol and isobutanol biosynthesis (engineered) |
| DENITRIFICATION-PWY  PWY-6662  PWY-7039  PWY-6748  PWY-7853  PWY-5742  P241-PWY  PWY-7527  PWY-7294  PWY-4361 | Nitrate reduction I (denitrification)  Superpathway of quinolone and alkylquinolone biosynthesis  Phosphatidate metabolism, as a signaling molecule  Nitrate reduction VII (denitrification)  6-hydroxymethyl-dihydropterin diphosphate biosynthesis V (*Pyrococcus*)  L-arginine degradation IX (arginine:pyruvate transaminase pathway)  Coenzyme B biosynthesis  L-methionine salvage cycle III  D-xylose degradation IV  S-methyl-5-thio- and α-D-ribose 1-phosphate degradation I |

| **Controls, CI (2 unique pathways)** | |
| --- | --- |
| P163-PWY | L-lysine fermentation to acetate and butanoate |
| PWY-5101 | L-isoleucine biosynthesis II |

| **Cancer survivors, no CI (4 unique pathways)** | |
| --- | --- |
| PWY-7279 | Aerobic respiration II (cytochrome c) (yeast) |
| PWY-6113  PWY-7317  PWY-5724 | Superpathway of mycolate biosynthesis  Superpathway of dTDP-glucose-derived O-antigen building blocks biosynthesis  Superpathway of atrazine degradation |

Abbreviations: CI: Cognitive impairment, dTP: Deoxythymidine diphosphate
