# Supplementary material for: Gut microbiome signatures predict cognitive impairment in older cancer survivors
Source: GeroScience. 2025 Nov 1;48(4):5575–96. doi: 10.1007/s11357-025-01917-x (PMC13575041; doi:10.1007/s11357-025-01917-x)
Supplement: Supplementary file 5 [file 11357_2025_1917_MOESM6_ESM.docx]

**Supplementary Table S5.** Increased and decreased pathways in cancer survivors compared to non-cancer controls further stratified based on cognitive function.

**Controls (no CI) vs. Cancer Survivors (no CI)**

| **Pathway** | | | **Pathway Description** | **log2(fold change)** | **-log_10_(p value)** |
| --- | --- | --- | --- | --- | --- |
| PWY-8190 | | L-glutamate degradation XI (reductive Stickland reaction) | | 3.5791 | 2.2542 |
| PWY-5188 | | Tetrapyrrole biosynthesis I (from glutamate) | | 0.3277 | 2.0000 |
| PWY-5690  PWY-7754 | | TCA cycle II (plants and fungi)  Bile acid 7 & α-dehydroxylation | | 0.8979  3.0028 | 1.7213  1.7036 |
| PWY-8134 | | Bile acid 7 & β-dehydroxylation | | 2.5746 | 1.5866 |
| PWY490-3 | | Nitrate reduction VI (assimilatory) | | 3.1770 | 1.3532 |
| PWY-7031 | | Protein N-glycosylation (bacteria) | | 3.7134 | 1.3454 |
| PWY-6167 | | Flavin biosynthesis II (archaea) | | -2.6331 | 1.3377 |
|  |  | | |  |  |

**Controls (CI) vs. Cancer Survivors (CI)**

| **Pathway** | | | **Pathway Description** | **log2(fold change)** | **-log_10_(p value)** |
| --- | --- | --- | --- | --- | --- |
| PWY-6527 | | Stachyose degradation | | 0.4102 | 1.4758 |
|  |  | | |  |  |

**Controls (no CI) vs. Controls (CI)**

| **Pathway** | | | **Pathway Description** | **log2(fold change)** | **-log_10_(p value)** |
| --- | --- | --- | --- | --- | --- |
| PWY-6630  PWY-6588  GLYCOLYSIS-E-D  PWY-7761 | Superpathway of L-tyrosine biosynthesis  Pyruvate fermentation to acetone  Superpathway of glycolysis and the Enter-Doudoroff pathway  NAD salvage pathway II (PNC IV cycle) | | | -0.6513  -1.0531  -0.6790  -0.4200 | 2.1253  2.0682  2.0417  1.7906 |
| PWY-7345  PWY-5130  PWY-8004  GLUCONEO-PWY  PWY-7211  POLYAMINSYN3-PWY  PWY-6293  PWY-821  PWY-6895 | | Superpathway of anaerobic sucrose degradation  2-oxobutanoate degradation I  Entner-Duodoroff pathway I  Gluconeogenesis I  Superpathway of pyrimidine deoxyribonucleotides de novo biosynthesis  Superpathway of polyamine biosynthesis II  Superpathway of L-cysteine biosynthesis (fungi)  Superpathway of sulfur amino acid biosynthesis (*Saccharomyces cerevisiae*)  Superpathway of thiamine diphosphate biosynthesis II | | -0.5078  -1.0854  -0.6108  -0.3217  -1.2356  -1.2746  -1.3571  -1.3929  -0.6119 | 1.7637  1.5863  1.5740  1.5706  1.5584  1.5067  1.4808  1.4734  1.4707 |

**Cancer Survivors (no CI) vs. Cancer Survivors (CI)**

| **Pathway** | | | **Pathway Description** | **log2(fold change)** | **-log_10_(p value)** |
| --- | --- | --- | --- | --- | --- |
| PWY-7761  PWY-7328  PWY-6588  PWY0-1297  PWY-6630 | NAD salvage pathway II (PNC IV cycle)  Superpathway of UDP-glucose-derived O-antigen building blocks biosynthesis  Pyruvate fermentation to acetone  Superpathway of purine deoxyribonucleosides degradation  Superpathway of L-tyrosine biosynthesis | | | -0.6903  -0.5248  -1.5671  -0.9776  -0.7019 | 2.8586  2.3715  2.1930  2.0503  2.0273 |
| UDPNAGSYN-PWY  PWY-7211  PWY-6545  PWY-7184  PWY0-1241  PWY-7210  PWY-5690  PWY0-1298  PWY-7198  P161-PWY  OANTIGEN-PWY  PWY4LZ-257 | | UDP-N-acetyl-D-glucosamine biosynthesis I  Superpathway of pyrimidine deoxyribonucleotides de novo biosynthesis  Pyrimidine deoxyribonucleotides de novo biosynthesis III  Pyrimidine deoxyribonucleotides de novo biosynthesis I  ADP-L-glycero-beta-D-manno-heptose biosynthesis  Pyrimidine deoxyribonucleotides biosynthesis from CTP  TCA cycle II (plants and fungi)  Superpathway of pyrimidine deoxyribonucleosides degradation  Pyrimidine deoxyribonucleotides de novo biosynthesis IV  Acetylene degradation (anaerobic)  O-antigen building blocks biosynthesis (*E. coli*)  Superpathway of fermentation (*Chlamydomonas reinhardtii*) | | -0.4742  -2.0940  -2.6805  -2.8106  -1.6471  -3.6241  -1.2773  -1.3308  -0.6060  -1.6912  -0.3032  -1.4040 | 2.0056  1.9111  1.8013  1.7818  1.6329  1.5702  1.4675  1.4573  1.4188  1.4126  1.3978  1.3205 |

Abbreviations: ADP: Adenine diphosphate, CI: Cognitive impairment, CTP: Cytidine 5′-triphosphate *E. coli*: *Escherichia coli*, NAD: Nicotinamide adenine dinucleotide, PNC: Pyridine nucleotide cycle, TCA: Tricarboxylic acid, UDP: Uridine diphosphate,
