# Supplementary material for: Gut microbiome signatures predict cognitive impairment in older cancer survivors
Source: GeroScience. 2025 Nov 1;48(4):5575–96. doi: 10.1007/s11357-025-01917-x (PMC13575041; doi:10.1007/s11357-025-01917-x)
Supplement: Supplementary file 6 [file 11357_2025_1917_MOESM7_ESM.docx]

**Supplementary Table S6.** Associations between significant taxa and metabolic pathways based on hierarchal correlation clustering.

| **Taxa and pathways** | **r value** | **p-value** |
| --- | --- | --- |
| ***Streptococcaceae***  PWY-5188  PWY-7328  PWY0-1241 | 0.2831  0.1850  0.2236 | 0.0005***  0.0239*  0.0061** |
| ***Erysipelotrichaceae***  GLUCONEO-PWY  GLYCOLYSIS-E-D  PWY-7761  PWY-8004    ***Oscillospiraceae***  PWY-8190  ***Streptococcus***  PWY-5188  PWY-7328  PWY0-1241  ***Tyzzerella***  PWY-6545  PWY-7184  PWY0-1241  PWY-7210    ***Ruminoccaceae* (unclassified)**  PWY-7761  PWY-5130  PWY-8190  PWY-7031  PWY-7328  ***Lawsonibacter***  PWY-5130  PWY-7031  ***Holdemania***  PWY-7031  ***Oscillibacter***  PWY-8190  ***Streptococcus thermophilus***  PWY-6630  PWY-6293    ***Firmicutes bacterium CAG 114***  GLYCOLYSIS-E-D  PWY-5130  PWY-8004  ***Streptococcus sp. A12***  PWY-6527  PWY-5188  PWY-7198  ***Clostridium citroniae***  PWY-5690  PWY490-3  ***Oscillibacter sp. CAG 241***  PWY-6167  ***Collinsella sp. CAG 289***  METH-ACETATE-PWY  ***Intestinibacter bartlettii***  PWY-6527  PWY-5188  METH-ACETATE-PWY  ***Ruminococcus sp. CAG 177***  GLYCOLYSIS-E-D  PWY-7345  PWY-7211  PWY-6293  PWY-821  PWY-6895  PWY0-1297  PWY-6545  PWY-7184  PWY0-1298  P161-PWY  PWY4LZ-257  ***Eubacterium sp. OM08_24***  PWY-6167  PWY-7211  PWY-6545  PWY-7184  PWY-7210  ***Firmicutes bacterium CAG 110***  GLYCOLYSIS-E-D  PWY-8004  PWY4LZ-257  ***Eggerthella lenta***  PWY-8134  PWY-7754  ***Bacteroides eggerthii***  PWY-5690  PWY-5130  ***Butyricimonas synergistica***  PWY-5690  PWY-6588  PWY-7211  PWY-821  PWY0-1298  PWY4LZ-257  ***Dialister sp. CAG 357***  PWY-6588  GLUCONEO-PWY  PWY-821  PWY-7211  PWY0-1241  PWY-7210  P161-PWY  PWY4LZ-257    ***Bacteroides vulgatus***  GLYCOLYSIS-E-D  PWYO-1297  P161-PWY  ***Lawsonibacter asaccharolyticus***  PWY-5130  PWY-7031  ***Clostridium scindens***  PWY-8190  PWY0-1241  ***Lachnospiraceae bacterium OF09 33XD***  PWY-8190  ***Holdemania filiformis***  PWY-7031  ***Dorea sp. CAG 317***  PWY-7031  ***Eggerthella sp. CAG 209***  PWY-6545  PWY-7184  PWY-7210  PWY-7198  ***Lactobacillus phage A2***  PWY-7754  PWY-5690  PWY-8190  PWY-8134  PWY0-1241  ***Lactococcus phage P335 sensu lato***  PWY-5188  PWY0-1241 | 0.2394  0.1877  0.1724  0.1853    0.1696  0.2908  0.1824  0.2312  0.2661  0.2654  0.1908  0.2807  0.1912  0.1994  0.2369  0.3448  0.1684  0.3091  0.2458  0.2380  0.1696  0.1901  0.1781  0.2164  0.2861  0.3144  0.1720  0.1631  0.1935  0.1984  0.2073  0.1871  0.1956  0.2187  0.1725  0.2199  0.2120  0.2511  0.1781  0.1899  0.2765  0.2412  0.1723  0.1657  0.2105  0.2948  0.3198  0.3730  0.1642  0.2327  0.2034  0.1717  0.1611  0.1836  0.2203  0.1779  0.5606  0.5620  0.2614  0.1611  0.1643  0.1856  0.1767  0.1767  0.1623  0.1683  0.1966  0.1620  0.2115  0.2609  0.1934  0.1966  0.1677  0.1829  0.1686  0.2211  0.2044  0.3091  0.2458  0.2433  0.2354  0.1867  0.2380  0.1688  0.2211  0.2203  0.2893  0.2146  0.2469  0.2100  0.1753  0.2478  0.1843  0.2372  0.2136 | 0.0033**  0.0219*  0.0355*  0.0237*  0.0387*  0.0003***  0.0260*  0.0046**  0.0010**  0.0011**  0.0197*  0.0005***  0.0195*  0.0148*  0.0036**  <0.0001****  0.0401*  0.0001***  0.0025**  0.0035**  0.0387*  0.0203*  0.0318*  0.0080**  0.0004***  <0.0001****  0.0360*  0.0469*  0.0180*  0.0153*  0.0112*  0.0223*  0.0168*  0.0074**  0.0354*  0.0071**  0.0095**  0.0020**  0.0298*  0.0204*  0.0006***  0.0030**  0.0357*  0.0434*  0.0100*  0.0003***  <0.0001****  <0.0001****  0.0454*  0.0043**  0.0129*  0.0363*  0.0496*  0.0250*  0.0070**  0.0299*  <0.0001****  <0.0001****  0.0013**  0.0497*  0.0453*  0.0235*  0.0311*  0.0311*  0.0479*  0.0402*  0.0163*  0.0484*  0.0096**  0.0013**  0.0181*  0.0163*  0.0409*  0.0255*  0.0399*  0.0067**  0.0124*  0.0001***  0.0025**  0.0028**  0.0038**  0.0226*  0.0035**  0.0396*  0.0067**  0.0069**  0.0003***  0.0086**  0.0024**  0.0102*  0.0325*  0.0023**  0.0245*  0.0036**  0.0089** |

R values were calculated using Pearson’s correlation coefficient.

*p < 0.05, **p < 0.01, **p < 0.001, ****p < 0.0001.
